# Fluoroquinolone-associated tendinopathy: a case report

**DOI:** 10.1186/1752-1947-1-55

**Published:** 2007-07-23

**Authors:** Wan-Fai Ng, Michael Naughton

**Affiliations:** 1 Department of Rheumatology, Ealing Hospital NHS Trust, Ealing, Southall, UB1 3UJ UK; 2 Musculoskeletal Research Group, Institute of Cellular Medicine, University of Newcastle, Newcastle NE2 4HH, UK

## Abstract

Fluoroquinolone-associated tendinopathy is well described. This adverse effect however does not appear to be widely known among medical practitioners. We hereby described a case of ciprofloxacin-associated tendinopathy for which the adverse drug reaction was not suspected initially and the patient was inappropriately reassured and incorrectly advised to complete the antibiotic course. Given the frequent use of fluoroquinolones in clinical practice and the potential for severe disability from tendon rupture, we consider it important to remind your readers of this uncommon but potentially devastating adverse drug reaction.

## Background

Fluoroquinolones are a family of broad-spectrum bactericidal antibiotics that are frequently used in the treatment of a wide range of infections. They are considered to be relatively safe and well tolerated. However, although it is well documented that fluoroquinolones predispose to tendinopathy, this adverse effect does not appear to be widely known among medical practitioners. Given the frequent use of fluoroquinolones in clinical practice and the potential for severe disability from tendon rupture, we consider it important to remind your readers of this uncommon but potentially devastating adverse drug reaction.

## Case presentation

A 42-year-old woman presented to the emergency department of a district general hospital with acute onset of generalised pain several hours after taking ciprofloxacin for presumed urinary tract infection. Clinical examination at the time was unremarkable. She was reassured and advised to finish the 5-day course of antibiotics. Her symptoms persisted after 3 weeks and she was referred for a rheumatological opinion.

She described feeling that the tendons of her hands, left knee and left ankle were "inflamed". She had morning stiffness lasting 30 minutes and the pain was worse at the end of the day. She had pain on walking and performing simple tasks with her hands. There was no joint swelling and she denied other symptoms. She used ibuprofen as required for symptom control.

Her past medical history included uterine fibroids and recurrent "cystitis" secondary to dystonic bladder awaiting further investigations. In addition to a busy job she pursued a wide range of sporting activities.

Examination of the hands revealed tenderness of the flexor and extensor tendons with poor grip secondary to pain. The patellar and Achilles tendons were tender to palpation but no swelling was detected. There was no swelling or tenderness of her peripheral joints. Laboratory investigations revealed normal inflammatory markers, renal and liver biochemistry and complete blood count. Autoimmune serology was negative. Ultrasound examination of her Achilles tendons showed no evidence of a tendon tear or rupture.

A diagnosis of ciprofloxacin-related tendinopathy was made. She was treated with ibuprofen as required and a graded exercise programme. Her symptoms improved after 3 weeks and completely resolved after 4 months.

## Discussion

The first case of fluoroquinolone-associated tendinopathy was reported in 1983 [1]. Since then, nearly 100 case reports have been published, many of which originate from France, due to the publicity generated in that country.

The incidence of this condition is not clear. A rate of 2.9 per 1000 prescriptions for tendinitis has been reported in one study [2] with an overall excess risk of 3.2 cases per 1000 patient years [3]. With prescription-event monitoring, a rate of 2.4 per 10,000 patients for tendinitis, and 1.2 per 10,000 patients for tendon rupture was found [4]. Risk factors include age over 60, corticosteroid therapy, sporting activity, history of musculoskeletal disorders, renal failure and diabetes [3,5,6].

The commonest presenting complaint of fluoroquinolone-associated tendinopathy is pain, typically of sudden onset. Other symptoms include swelling, warmth, tenderness, erythema or itchiness over tendon sites and functional disability. Bilateral involvement is common. Clinical findings include tendon swelling, thickening or oedema [5,6]. The commonest tendon affected is the Achilles tendon, but other tendons can also be affected [5,6]. Up to 50% of patients may develop tendon rupture with nearly one-third of these in patients taking long-term corticosteroid therapy [5]. It is noteworthy that up to half of the tendon ruptures occurred without warning [5,6].

The latency period between the start of fluoroquinolone treatment and symptom ranges from a few hours to a few weeks, with a mean of 6–10 days. Most patients recover within 2 months after cessation of therapy, but over a quarter of patients suffer with persistent pain and disability [6]. Other musculoskeletal side-effects of quinolones include arthropathy, myalgia and myopathy.

The diagnosis is usually clinical. Ultrasound and magnetic resonance imaging of the affected tendons is helpful in identifying tendon tear or rupture.

Management is largely symptomatic with discontinuation of the offending antibiotics, use of analgesics/anti-inflammatory medications and physiotherapy. Rest and splinting may be necessary. Tendon rupture often requires surgical repair.

The pathogenesis of fluoroquinolone-related tendinopathy has not been established. Several mechanisms such as direct toxic effect [7,8] and ischaemic injury [9] have been proposed.

This case highlights the importance of suspecting possible drug reaction when there is a clear temporal relationship between treatment and symptoms, regardless of how commonplace the medication concerned. Indeed, the drug information on quinolones in the British National Formulary (BNF) includes the CSM advice on tendon damage associated with quinolone use [10]. It advises immediate discontinuation of treatment if tendinitis is suspected.

In summary, we have presented a case of ciprofloxacin-associated tendinopathy and highlighted the importance of recognising a potentially disabling complication of this common antibiotic.

## Conclusion

Fluoroquinolone-associated tendinopathy should be considered in patients with musuloskeletal symptoms associated with recent use of fluoroquinolones.

## Competing interests

The author(s) declare that they have no competing interests.

## Authors' contributions

WFN was responsible for conceptualising and drafting of the manuscript, MN was responsible for critically reviewing the manuscript and the care of the patient of the index case. Both authors read and approved the final manuscript.
